# A Lower Limb Rehabilitation Robot in Sitting Position with a Review of Training Activities

**DOI:** 10.1155/2018/1927807

**Published:** 2018-04-01

**Authors:** Trinnachoke Eiammanussakul, Viboon Sangveraphunsiri

**Affiliations:** Department of Mechanical Engineering, Faculty of Engineering, Chulalongkorn University, Bangkok, Thailand

## Abstract

Robots for stroke rehabilitation at the lower limbs in sitting/lying position have been developed extensively. Some of them have been applied in clinics and shown the potential of the recovery of poststroke patients who suffer from hemiparesis. These robots were developed to provide training at different joints of lower limbs with various activities and modalities. This article reviews the training activities that were realized by rehabilitation robots in literature, in order to offer insights for developing a novel robot suitable for stroke rehabilitation. The control system of the lower limb rehabilitation robot in sitting position that was introduced in the previous work is discussed in detail to demonstrate the behavior of the robot while training a subject. The nonlinear impedance control law, based on active assistive control strategy, is able to define the response of the robot with more specifications while the passivity property and the robustness of the system is verified. A preliminary experiment is conducted on a healthy subject to show that the robot is able to perform active assistive exercises with various training activities and assist the subject to complete the training with desired level of assistance.

## 1. Introduction

Robots for rehabilitation have gained more attentions in many research due to some benefits over conventional therapy by physiotherapists. For example, robots for locomotion training on a treadmill primarily aim to replace physical demand of the therapist labor because the task is ergonomically unfavorable and tiring [1]. Without physical burden, numbers of repetition and duration of the training session can be increased [2–4]. While the performance of a therapist could vary from day to day [1] and intervention techniques by expert and unexperienced therapists are different [5, 6], a robot follows the certain control algorithm and provides systematic intervention to the patient. Moreover, robots are able to obtain and record data such as position, velocity, interaction force, or biosignal with various kinds of sensors. This quantitative data can be used for further offline analysis, which leads to objective evaluation of the patient's recovery [3, 4], or even used for adapting robot's behavior corresponding the patient's current condition. Rehabilitation robots are also able to perform different types of exercises and varieties of movement [2, 7, 8]. Moreover, the robot can be implemented with games [9] or virtual reality system [10] in order to promote active participation of the patient. Robots for stroke rehabilitation have shown their effectiveness in many clinical trials worldwide.

The lower limb rehabilitation robots can be categorized into 2 types according to exercise posture [11]. The first type is a robot for training in sitting/lying position which benefits the patient who still suffers from muscle weakness and cannot stand or walk safely [5, 12]. By excluding concern of balance, the patient may be more independent and able to focus on the training [13]. This kind of robot allows the patient to strengthen muscle, develop endurance, and increase joint mobility and movement coordination [4]. Another type of the robot is for training while standing/walking. The gait training robot in literature was developed to train either over ground or on a treadmill with a body weight support mechanism. However, the gait training robot is only suitable for the patient that has adequate endurance and ability to stand [14].

Training modalities used in robots for stroke rehabilitation are often divided into four groups, namely, passive, active assistive, active, and active resistive exercises [15]. Exercises may be prescribed to a stroke patient corresponding to the stage of recovery [3] and the muscle tone [2, 16], which can be graded according to the muscle contraction observability, ability to move a limb against gravity and external resistance [17]. In the preliminary stage, the patient with very low muscle strength should conduct passive training, for example, moving the patient's limb along a predefined trajectory by another person or an exercise device known as continuous passive machine (CPM). Passive exercise could improve movement ability, maintain range of motion, and reduce muscle atrophy. In the intermediate stage, the patient with some degree of muscle strength should perform active assistive, active, or active resistive exercise. Active assistive exercise allows the patient to move the limb by himself with assistance provided by another person when needed. Active exercise allows the patient to move by his own effort without external assistance and resistance. In active resistive mode, the robot provides force opposing the movement of the patient. This exercise aims to strengthen muscle of the patient who is already able to move his limb over the full range of motion. Different motion and amount of resistive force can be applied to achieve a variety of strengthening training such as isometric, isotonic, and isokinetic exercise. There are many types of force that could be applied to the patient as resistance. Resistive exercise in conventional rehabilitation can be done against weight, elastic bands, in the pool, or by an exercise machine [14]. In the advanced rehabilitation stage, the objective of the exercise is to regain function related to activities in daily living such as balancing and gait training.

Apart from training modalities, training activities also have to be selected appropriately to an individual. Because the lower limb rehabilitation robots in sitting/lying position get rid of the stability concern, the robots are able to perform a variety of training activities. It is interesting to study how these training activities are selected and what are benefits of each training activity for a stroke patient. This knowledge would be useful in developing robots suitable for stroke rehabilitation.

This article focuses on the lower limb rehabilitation robots for training in sitting/lying position. In Section 2, the training activities performed by this type of robot are reviewed. Description of the robot is shown in Section 3. The control system and the impedance control law proposed in our previous work [18] for active assistive exercises are discussed in Section 4. Experiments conducted to study the performance of the system are also presented in this section. The experiment by a healthy subject in Section 5 demonstrates the robot behavior while performing training activities with many levels of assistance. Finally, the conclusion of the research article is made on Section 6.

## 2. Review of Training Activities of Robots for Stroke Rehabilitation

Training activities performed by lower limb stroke rehabilitation robots in sitting/lying position are summarized in Table 1. Types and actuated degrees of freedom of the robots are also reported since they are corresponding to the training activities. Foot robotic orthoses and footplate-based end-effector robots are able to actuate only at ankle joint and foot section. End-effector robots (nonfootplate-based type) are usually able to perform movements involving hip, knee, and ankle joints in the sagittal plane. On the other hand, exoskeletons whose structure and joint axes aligned with those of human body are able to perform the movement of a single joint or multiple joints.

Robots in lower limb rehabilitation after stroke are developed with different concepts of the training. Some robots are able to perform a certain training activity and modality. Anklebot [19] is able to train ankle joint with active assistive training modality while the lower limb paediatric therapy device by Chrif et al. [20] was designed for leg press exercise performing with active resistive training modality. Besides, some robots can perform many training activities but with a certain training modality. For example, the horizontal lower limb rehabilitation training robot by Guo et al. [5] is able to train lower limbs in six actions according to the traditional Chinese medicine technique with passive training modality, whereas ViGRR [21] can perform gait trajectory following and leg press exercise with active resistive training modalities. Moreover, some robots are able to perform only one training activity but with various kinds of training modality. MOTOmed [22] can perform cycling exercise with passive, active assistive, and active resistive training modalities while Vi-RABT [12] can train ankle joint with active assistive and active training modalities. The other robots are able to perform many training activities and modalities. The ankle rehabilitation robot by Yoon et al. [7] is able to train ankle joint or perform gait trajectory following at the ankle joint with passive, active assistive, and active resistive training modalities. Physiotherabot [2] is able to perform many training activities at hip and knee joints with passive, active assistive, and active resistive training modalities. Lower limb rehabilitation robot in our previous research [18] can be used for therapeutic exercises at hip, knee, and ankle joints. Because the robot structure is exoskeleton, it can perform both single- and multiple-joint training. The desired trajectory of the robot can be easily customized. Therefore, range, pattern, and speed of the movement can be arbitrarily adjusted to suit with the patient condition. It is also able to perform passive, active assistive, and active resistive training modalities. The robot is designed for versatile training for a stroke patient in sitting position.

According to Table 2, training activities can be categorized as single-joint training and multiple-joint training. The single-joint training involves the movement of a specific joint (hip, knee, or ankle joint) in one or several degrees of freedom. It can be performed by foot robotic orthoses, footplate-based end-effector robots, and exoskeletons. On the other hand, the multiple-joint training consists of the simultaneous movement of several joints for performing exercises such as leg press, cycling, and gait trajectory following. Some robots are able to perform a customized movement by using recorded data (e.g., position, velocity, and interaction force) obtained during the robot teaching session by a physiotherapist. The multiple-joint training can be conducted by end-effector robots and exoskeletons.

Single-joint training is usually selected for range of motion exercise which can be performed with passive, active assistive, or active training modalities. In addition, single-joint training is also chosen when improvement of functional ability of a specific joint is required. For example, the ankle joint is targeted for the training by some rehabilitation robots because stroke patients are usually unable to activate dorsiflexor muscle to lift the foot up. This problem leads to walking impairments of the patients such as toe dragging in the swing phase and foot slapping in the heel strike phase. Besides, the patients might have excessive inversion which causes lateral instability in the stance phase of the gait [19]. Anklebot and Vi-RABT apply active assistive training modality to provide assistance to a patient while using the robots to move a cursor in computer games. The benefits of the training are supported by results of clinical trials on chronic stroke patients with Anklebot. It was shown that the patients had better motor control (increased targeting accuracy and faster and smoother movements) and walking ability (increased walking velocity, durations of paretic single support, and nonparetic step length which could be a result from greater push-off of the paretic foot) [23–25].

There are varieties of exercise that involve training of multiple joints such as leg press, cycling, gait trajectory following and customized movement. The developers of the robots selected one or several kinds of these exercises to achieve different aspects of the stroke recovery.

Leg press exercise is extensively used in sport and neuromuscular rehabilitation. It aims to strengthen muscles across multiple joints of the lower limbs in sitting/lying position [26]. This exercise is able to activate leg muscles in a similar level compared to bodyweight exercises such as chair rise and hip thrust [27]. Moreover, it is found that chronic stroke patients not only gain strength on both affected and nonaffected legs but also have improvement in balance, walking ability, and functional performance [28, 29].

Cycling is an alternative exercise to walking for stroke patients who have difficulty in maintaining balance and independent gait [30]. It provides continuous repetitions of movement which promotes coordination of muscle synergies. Its kinematic pattern is also similar to walking as it requires flexion and extension of hip, knee, and ankle joints as well as activation of antagonist muscles in alternating and coordinated manner. In addition, because the range of motion in cycling is greater than that in walking, cycling could help maintaining functional range of motion as a preparation for gait training in the future [31]. It was found that the stationary cycling training is able to enhance dynamic balance, muscle strength, and walking ability of chronic stroke patients [30]. MOTOmed, which was specifically designed for cycling exercise, had been used in clinical trials on stroke patients. It was found that stroke patients who performed resistive exercise with the device had improvement in walking distance in 2 and 6 minutes walking test, increase in comfortable speed, and lower time spent on “Up & Go” test [22].

Walking is a functional task of lower limbs and the goal of rehabilitation. However, the task consists of complex movement that requires force generation for body weight support, coordination, and weight shifting [31]. Gait training of a stroke patient who still suffers from muscle weakness demands great physical effort from both the patient and several physiotherapists. Therefore, duration and numbers of repetition in a gait training session in an upright position might not be enough to gain effective rehabilitation outcome [1]. Some robots are able to perform gait training for stroke patients in sitting/lying position. These robots recorded gait trajectories from healthy subjects to create a reference data for training stroke patients. The ankle rehabilitation robot by Yoon et al. performs isokinetic exercise by following ankle and foot (metatarsophalangeal joint) reference trajectory. On the other hand, ViGRR implements resistive exercise against virtual damping and inertia to interact with the patient during the gait trajectory following task.

Because physical characteristics may differ among stroke patients and from day to day, the training should be customized individually at the beginning of each training session. Physiotherabot was developed to train a stroke patient with any movement pattern taught by a physiotherapist. Once the movement is recognized, the robot will train a stroke patient with that movement as if the training is performed by a physiotherapist.

## 3. Lower Limb Rehabilitation Robot

**Figure 1:**
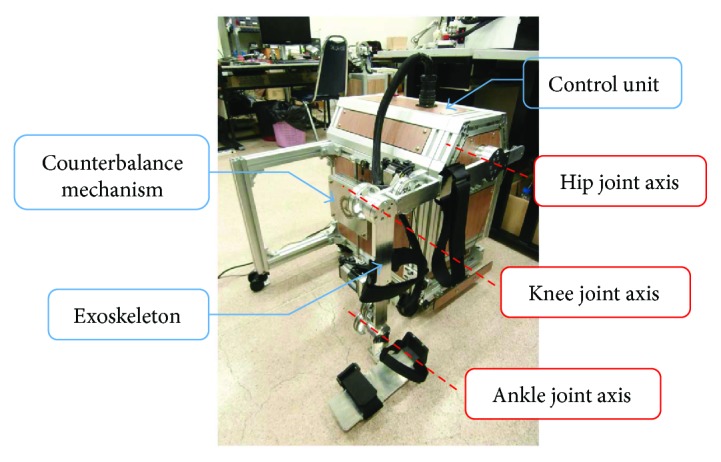
Lower limb rehabilitation robot in sitting position consisted of an exoskeleton (with hip, knee, and ankle joints), the counterbalance mechanism, and the control unit.

The lower limb rehabilitation robot in this project is developed for movement training in sitting position. It aims to be used by patients who have severe hemiparesis condition. These patients are not comfortable to use typical treadmill training devices at the beginning of training activities. The sitting position robot is more preferable especially at the beginning state of training. The lower limb rehabilitation robot in this study as shown in Figure 1 consists of a powered exoskeleton, a counterbalance mechanism, a control unit, and a monitor screen (not shown in the figure).

The exoskeleton is able to move in the sagittal plane at hip, knee, and ankle joints. The hip joint allows 45° flexion and 0° extension. The knee joint is able to move in the range of 110° flexion and 0° extension. The ankle joint permits 20° dorsiflexion and 45° plantar flexion. These ranges of motion are ensured by mechanical stoppers placed at the end of the joint movement range.

**Figure 2:**
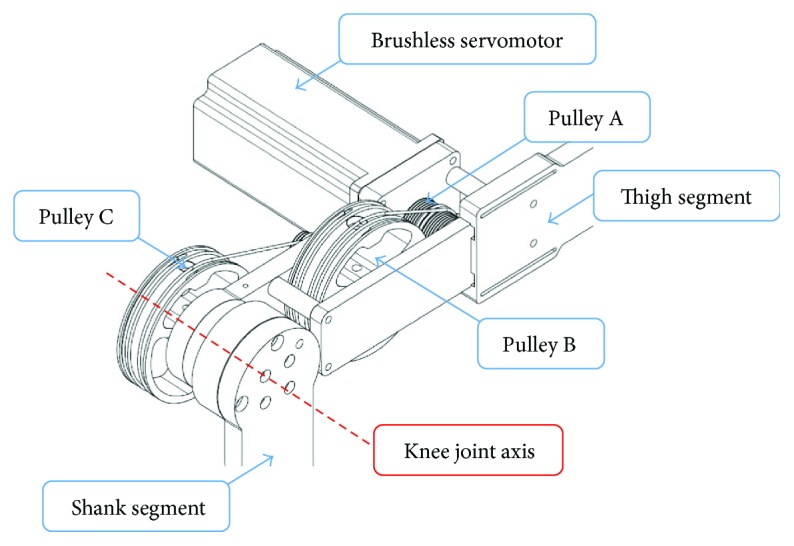
Cable transmission mechanism of the knee joint.

Figure 2 illustrates components of the cable transmission mechanism of a robot joint (the knee joint is shown, e.g.). The mechanism is actuated by a brushless servomotor (SANYO DENKI). Sizes of the motors are 400 W for hip and knee joints and 200 W for the ankle joint. Specifications of the motors are provided in Table 3. The pulley A, which is mounted at the end of the motor shaft, drives the pulley B via cable. The second stage of the cable transmission is done by the shaft connected to the pulley B. Another end of this shaft works as a small pulley for driving the pulley C via cable. With the shaft connected between the pulley C and the shank segment, the shank segment rotates about the knee joint axis with respect to the pulley C.

The torque requirements (maximum torque) of hip and knee joints are considered when lower limbs stretch out in sitting position while the torque requirement for the ankle joint is considered at neutral sitting position. According to anthropomorphic data [32], for a human with 100 kg weight and 180 cm height, torque requirements for hip, knee, and ankle joints are 67.331, 18.598, 1.945 N·m, respectively. For the robot joint design, transmission ratios of hip, knee, and ankle joints are chosen so that continuous torque provided by the robot joints is sufficient for the torque requirements. Specifications of the robot joints are shown in Table 4.

To achieve backdrivability of the robot joints, the inertia of the corresponding robot segment must be lower than the reflected motor inertia (the product of the square of the transmission ratio and the inertia of the motor) [33]. The ranges of inertia of thigh, shank, and foot segments according to their minimum and maximum lengths are given in Table 5. It can be noticed that the reflected motor inertias of hip, knee, and ankle joints are always lower than the moments of inertia of thigh, shank, and foot segments about their proximal joint axes, respectively. Therefore, it can be concluded that the robot joints are backdrivable.

The counterbalance mechanism is designed to reduce effects of the gravitational load due to robot's weight. The mechanism consists of vertical guide rods, linear bearings, a 12 kg mass, and a cable which wraps around a series of idlers to link the thigh segment of the exoskeleton and the 12 kg mass together. The guide rods and idlers are mounted on the control unit. The weight of the 12 kg mass generates counterbalance moment about the hip joint whose magnitude corresponds to the hip joint angle in order to counteract the moment due to robot's weight. With this counterbalance mechanism, the torque requirement of the hip joint transmission mechanism is reduced up to 20.7 N·m.

Both the exoskeleton and the counterbalance mechanism are installed on the control unit as a single platform. The control unit also contains a DC power supply, a computer unit, a data acquisition card, motor amplifiers, an emergency stop button, and other electronic devices.

## 4. Control System

The lower limb rehabilitation robot is developed to train a subject with various training activities and modalities. Control algorithm for each training modality had been introduced in the previous work [18]. In this study, the control algorithm for active assistive exercise is described in more detail.

### 4.1. Control Strategy

In active assistive exercise, a patient moves his limb in desired movements. The assistance on the patient's limb exerted by a physiotherapist will be given as much as necessary to achieve the task and only when needed.

Modern rehabilitation robots have realized this intervention technique, which is usually called the “assist-as-needed” control strategy, into their controllers. One of the most popular controllers is an impedance controller. This controller simulates the interaction between human and a robot with a function between force and kinematic variables (position, speed, and acceleration). The robot will interact to the environment (which is human, in this case) as if it is connected to virtual mechanical components such as springs, dampers, and masses. Since the characteristics of the human-robot interaction is controlled rather than position, the impedance controller allows some degree of position error and does not enforce the movement of the robot to follow the exact reference trajectory. This allows both spatial and temporal variability of the movement which does not only improve motor coordination and walking ability [34] but also promote active participation of the patient [3, 35]. Both variability and active participation are important factors for motor recovery as they provoke neuroplasticity and motor learning [35–37].

### 4.2. Control Architecture

**Figure 3:**
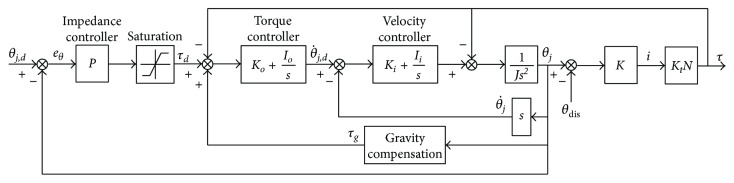
Block diagram of control algorithm for active assistive exercise.

The control algorithm for each joint of the robot as shown in Figure 3 consists of 3 cascaded loops which are outer, middle, and inner loops. The outer control loop is implemented with the impedance controller whose control law (*P*) is shown in (1). It establishes the relationship between joint angle error (*e*_*θ*_) and the magnitude of desired torque (*τ*_*d*_). In literature, many impedance control laws for rehabilitation robots were nonlinear such as Gaussian [12], polynomial [35, 38], or exponential [39] function. With a nonlinear impedance control law, low desired torque is generated for small position error but the magnitude of the desired torque increases with higher rate compared to the change of position error. This controller characteristic encourages a patient to move voluntarily if the position error is within acceptable tolerance. However, these control laws usually consist of one control gain. This could limit how the magnitude of desired torque changes with respect to position error. Therefore, the impedance control law developed for the robot in this research has two control gains *K*_1_ and *K*_2_, so the relationship between joint angle error and desired torque can be defined with more specifications. The procedure to select proper control gains will be presented in the next section of the article. Moreover, the impedance control law also considers the magnitude of desired force due to joint velocity error as represented by the last term in (1). The control gain *K*_*d*_ determines the magnitude of the damping force which could reduce oscillation of the human-robot interaction. It is noticed that the impedance controller in (1) is similar to a PD controller.

(1) $$\begin{array}{c} \tau_{d}=K_{1}\left(\exp\left(K_{2}\left|\theta_{j,d}-\theta_{j}\right|\right)-1\right)\mathrm{sgn}\left(\theta_{j,d}-\theta_{j}\right)+K_{d}\;d/dt\left(\theta_{j,d}-\theta_{j}\right). \end{array}$$

The saturation function is applied after the impedance control law to limit the maximum assistance force to be generated by the robot. The required gravitational torque (*τ*_*g*_) is also added to the desired torque to cancel the load at the robot joint due to robot's weight. For the hip joint, the required gravitational torque is reduced by the moment generated by the counterbalance mechanism. The resultant control signal is used as the torque reference for the middle control loop. A PI controller with control gains *K*_*o*_ and *I*_*o*_ is used to ensure perfect torque tracking.

The output of the torque controller in the middle control loop is used as the reference signal for the inner control loop. Another PI controller with control gains *K*_*i*_ and *I*_*i*_ is implemented to generate control signal to the motor driver in order to actuate a robot joint with inertia of *J*. The encoder mounted on the motor shaft measures the motor position. It can be used to estimate the position of the robot joint (*θ*_*j*_) by dividing the motor position by the total transmission ratio (*N*) of the robot joint. The velocity of the robot joint $\left({\dot{\theta }}_{j}\right)$ is differentiated from the estimated joint position.

Motor current (*i*) measured by the motor driver is detected due to the elastic force from the transmission mechanism. The magnitude of the elastic force is the product of the mechanism stiffness *K* and position difference between angle of the robot joint and angle at the load side that might be disturbed by an environment (*θ*_dis_). The joint torque is obtained from the motor current multiplied by the torque constant (*K*_*t*_) of the motor and the total transmission ratio of the joint.

By viewing the impedance controller as a PD controller, *P* becomes *K*_*d*_*s* + *K*_*p*_. The open loop transfer function of the system is derived as

(2) $$\begin{array}{c} \frac{\tau }{e_{\theta }}=\frac{{NKK}_{t}\left(K_{d}s+K_{p}\right)\left(K_{i}s+I_{i}\right)\left(K_{o}s+I_{o}\right)}{{Js}^{4}+K_{i}s^{3}+\left(I_{i}+{NKK}_{t}+{NKK}_{t}K_{i}K_{o}\right)s^{2}+{NKK}_{t}\left(K_{i}I_{o}+K_{o}I_{i}\right)s+{NKK}_{t}I_{i}I_{o}}. \end{array}$$

From (2), the system is strictly stable since the coefficient of the denominator is positive. Moreover, it can be noticed that the relative degree of the system is 1. Therefore, the phase shift of the system in response to sinusoidal inputs is always less than 90 degrees such that the Nyquist plot of (2) lies entirely in the right half complex plane. With these characteristics, it can be concluded that the system is strictly stable or passive [40]. This property implies that the system cannot output more energy than that was input into the system. In other words, stability of the interaction between the robot and an environment is guaranteed.

### 4.3. Control Gain Selection Procedure

The magnitude of the desired torque due to joint angle error (*τ*_*d*,*e*_*θ*__) is defined by the first term in the impedance control law (1)

(3) $$\begin{array}{c} \tau_{d,e_{\theta }}=K_{1}\left(\exp\left(K_{2}e_{\theta }\right)-1\right), \end{array}$$

where *e*_*θ*_ = |*θ*_*j*,*d*_ − *θ*_*j*_| is the magnitude of error between desired and actual joint position. It is noted that the function sgn(*e*_*θ*_) shown in (1) is for determining the direction of the desired torque, so it is omitted in (3).

Differentiating (3) with respect to position error, the rate of change of desired torque is obtained

(4) $$\begin{array}{c} \frac{d\tau_{d,e_{\theta }}}{{de}_{\theta }}=K_{1}K_{2}\exp\left(K_{2}e_{\theta }\right). \end{array}$$

The initial rate of change of desired torque (by setting *e*_*θ*_ = 0) is

(5) $$\begin{array}{c} {\left.\frac{d\tau_{d,e_{\theta }}}{{de}_{\theta }}\right|}_{e_{\theta }=0}=K_{1}K_{2}, \end{array}$$

so

(6) $$\begin{array}{c} K_{2}=\frac{1}{K_{1}}\left({\left.\frac{d\tau_{d,e_{\theta }}}{{de}_{\theta }}\right|}_{e_{\theta }=0}\right). \end{array}$$

Substituting (4) into (3) yields

(7) $$\begin{array}{c} \tau_{d,e_{\theta }}=K_{1}\left[\exp\left(\frac{e_{\theta }}{K_{1}}\left({\left.\frac{d\tau_{d,e_{\theta }}}{{de}_{\theta }}\right|}_{e_{\theta }=0}\right)\right)-1\right]. \end{array}$$

By specifying the maximum desired torque generated by the controller (*τ*_*d*,*e*_*θ*__^max^)and the maximum allowable position error (*e*_*θ*_^max^), it is found that

(8) $$\begin{array}{c} \tau_{d,e_{\theta }}^{\max}=K_{1}\left[\exp\left(\frac{e_{\theta }^{\max}}{K_{1}}\left({\left.\frac{d\tau_{d,e_{\theta }}}{{de}_{\theta }}\right|}_{e_{\theta }=0}\right)\right)-1\right]. \end{array}$$

If the initial rate of change of desired torque is known, the control gain *K*_1_ can be obtained by solving (5) numerically. Next, the control gain *K*_2_ can be calculated from (4).

### 4.4. Effects of the Impedance Controller Gains on the Robot Response

**Figure 4:**
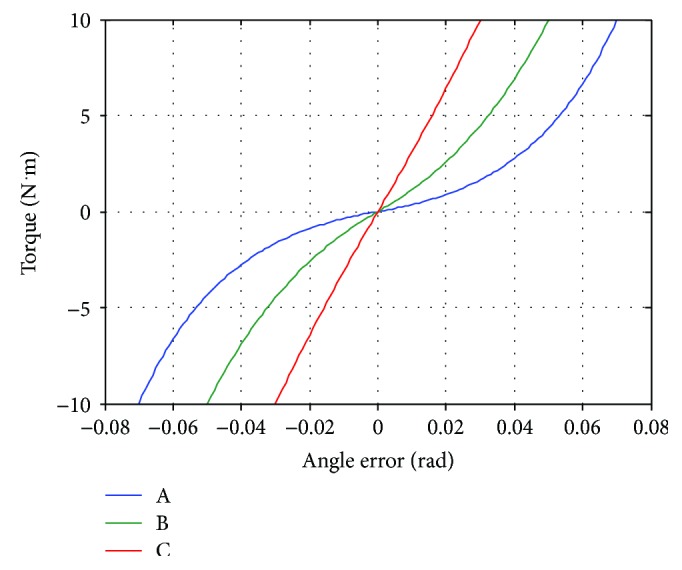
Relationship between angle error and torque generated by controllers A, B, and C of the ankle joint.

To study the effect of the nonlinear relationship between joint angle error and desired torque in (1). The ankle joint of the robot is tested with three sets of control gains as shown in Table 6. The control gains *K*_1_ and *K*_2_ are chosen so that the controller generates the maximum ankle torque (10 N·m) at joint angle errors of 0.03, 0.05, and 0.07 rad with different initial rate of change of desired torque (*K*_1_*K*_2_) as seen in Figure 4. The control gain *K*_*d*_ is the same for the controllers A, B, and C.

The objective of this experiment is to investigate the response due to the disturbance torque at robot's ankle joint which is implemented with controllers A, B, and C. During this experiment, no human subject is included and the disturbance torque is generated in robot's program by adding it before the torque control loop. The desired joint angle is always fixed at zero while the magnitude of the disturbance torque changes over time. Its magnitude increases from zero to the maximum value in 1 second. The maximum disturbance torque is hold for another second. Then, the magnitude decreases from the maximum value to zero in 1 second and is kept at zero until the end of each tests. The maximum magnitude of the disturbance torque is chosen as 1, 4, 7, and 10 N·m. This experiment simulates the circumstance when a human subject performs a movement training with the robot. At first, the subject gradually moves out of the desired path, stays at some position errors, and finally gets back to the desired path. The disturbance torque on robot's controller is caused from the position deviation from the desired path.

**Figure 5:**
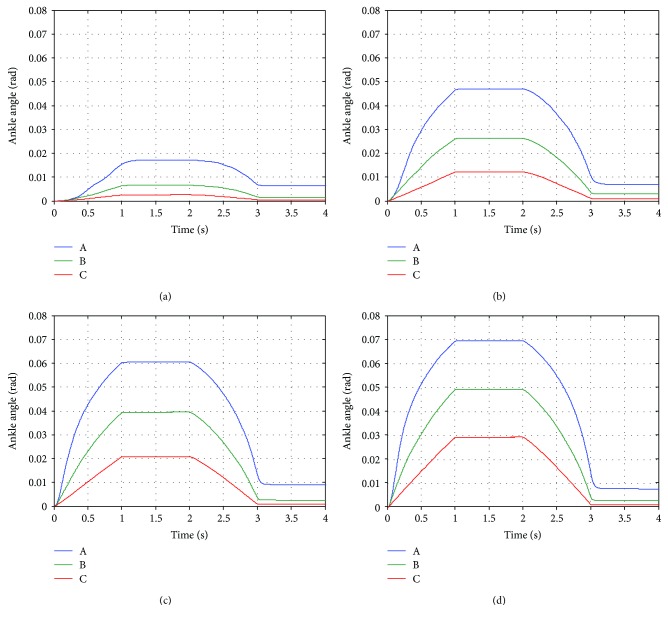
Response of robot's ankle joint implemented with the controllers A, B, and C as a result from the disturbance torque with maximum magnitude of (a) 1.0 N·m, (b) 4.0 N·m, (c) 7.0 N·m, and (d) 10.0 N·m.

Figure 5 shows the ankle position of the robot during the experiment with the controllers A, B, and C. Generally, it could be seen that the controller A always produces the highest angle error (deviation from the desired angle which is zero in this experiment) while the controller C generates the smallest angle error. The higher the magnitude of the disturbance torque, the higher the controllers produce angle error. During the first second, the controller A produces angle error which increases with varying rate as the magnitude of the disturbance torque is rising. On the contrary, the controller C creates angle error almost proportional to the magnitude of the disturbance torque. This difference originates from the relationship between angle error and torque generated by the controllers. As seen from Figure 4, the slope of the relationship of the controller A is very different at low and high angle error while the slope of the relationship of the controller C is almost constant. Therefore, with the same amount of change in the magnitude of the disturbance torque, the angle error produced by the controller A changes much faster than that by the controller C. The varying rate of angle change can also be found in the experiment with the controller B too, but the change is not as obvious as the controller A. While the magnitude of the disturbance torque is at the maximum value, the ankle angle in each case is constant. It can be noticed from Figure 5(d) that the angle error almost reached the certain error used in the controller design (0.07, 0.05, and 0.03 rad for the controllers A, B, and C). When the magnitude of the disturbance force is decreasing from its maximum value, the trends of the angle change by each controllers are also the same as in the first second of the experiment. After the magnitude of the disturbance torque reaches zero, it could be seen that the ankle position does not converge to zero. The remaining angle error is highest in the experiment with the controller A and lowest in the experiment with controller C. It is found that torque generated by these controllers is only around −0.3 N·m (not shown in Figure 5). Since friction in the robot mechanism is minimized by the robot design and the manufacturing of the robot parts, it is believed that the remaining angle errors are mainly due to the imperfect gravity compensation.

It can be concluded from this experiment that both control gains *K*_1_ and *K*_2_ determine how the robot responds to external torque. In order to obtain desired interaction between a human and the robot, these control gains should be selected appropriately. They can be calculated by using specified initial rate of change of the desired torque and maximum allowable angle error and torque. As demonstrated by the experimental results, the initial rate of change of desired torque defines the robot response at low angle error while the maximum angle error and torque determine the limit of the robot response. Therefore, if high angle error is allowed during the movement training, low initial rate of change of desired torque and high maximum angle error should be chosen. For more strict movements, high initial rate of change of desired torque and low maximum angle error should be selected. The maximum torque can be set according to the maximum capacity of the robot actuator or the amount of assistance required by an individual patient for a movement training. In the rest of the article, the impedance control gains of hip, knee, and ankle joints are selected with the same criterions used in this section. However, the maximum torque of hip and knee joints is 50 and 20 N·m, respectively. The controllers A, B, and C are named as low, medium, and high assistance controllers, respectively.  Table 7 summarizes the impedance control gains of the robot joints in each mode.

### 4.5. Robot Response due to External Impact Force

In Section 4.2, it was shown that the robot controller is passive. This property ensures the stability of the interaction between the robot and an environment. In this experiment, the robot is tested under impact force to verify the system's robustness in the sense of withstanding external impacts. During the experiment, there is no human subject worn the exoskeleton. The impact force is applied at the foot segment of the exoskeleton while it is fixed at a certain position (at hip, knee, and ankle angle of 0.703, 0.097, and 0.0 rad, resp.). The intensity of the impact force is high enough to reach the torque limit of at least one of the robot joints within a short period of time (torque limits for hip, knee, and ankle joints are 50, 20, and 10 N·m, resp.). The impedance control gains used in this experiment are referred to Table 7.

**Figure 6:**
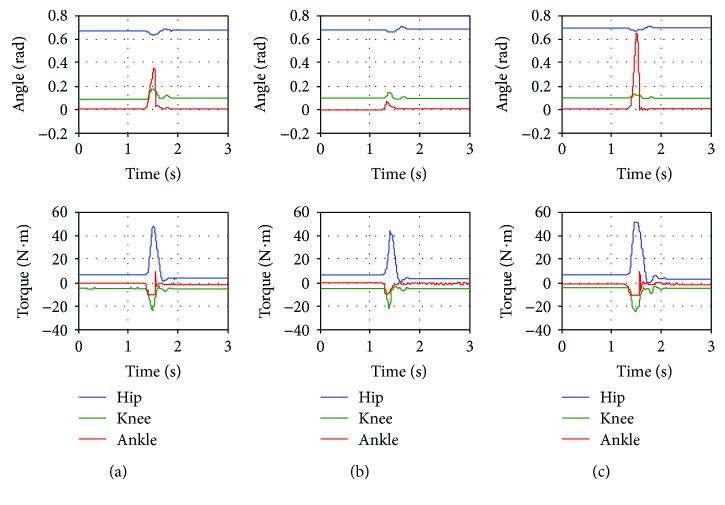
Response of the robot due to external impact force with control gains for (a) low assistance mode, (b) medium assistance mode, and (c) high assistance mode.

As seen in Figure 6, during the impact, hip, knee, and ankle angles deviate from the desired fixed position. Some robot joints generate torque at their maximum limit during the impact. Even though, the robot joint torque reaches the maximum limit as illustrated by flat peaks of torque signals, the robot joints finally get back to the desired position after few oscillations. This experiment has shown that the system is robust to external large impact force. Moreover, it confirms the stability of the system when interacting to an environment.

## 5. Experiment by a Healthy Subject with Various Training Activities

### 5.1. Method

**Figure 7:**
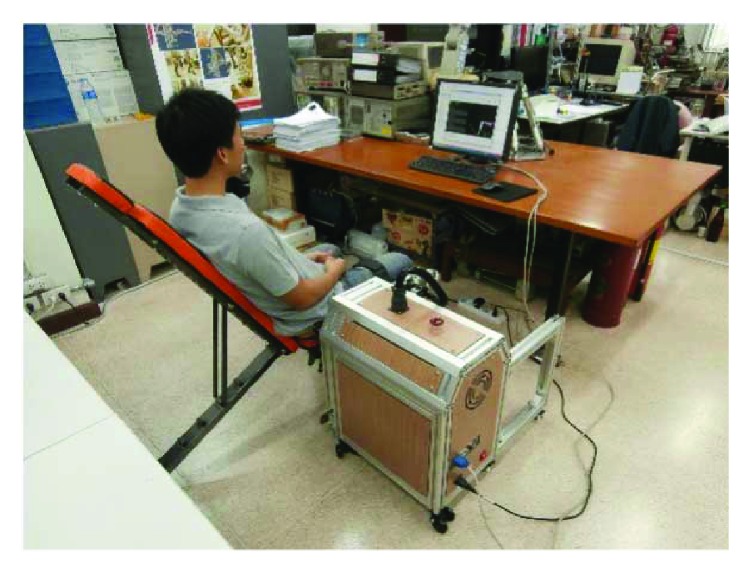
Training a subject with the robot.

To train a human subject with the robot as shown in Figure 7, the subject has to sit on a chair with an adjustable inclination backseat next to the control unit. Next, lengths of the exoskeleton segments are adjusted so that the robot fits on the subject's leg where the axes of hip, knee, and ankle joints of the subject are aligned with robot joint axes. Then, Velcro straps are used to fasten the subject's leg and the exoskeleton together at thigh, shank, and foot segments.

**Figure 8:**
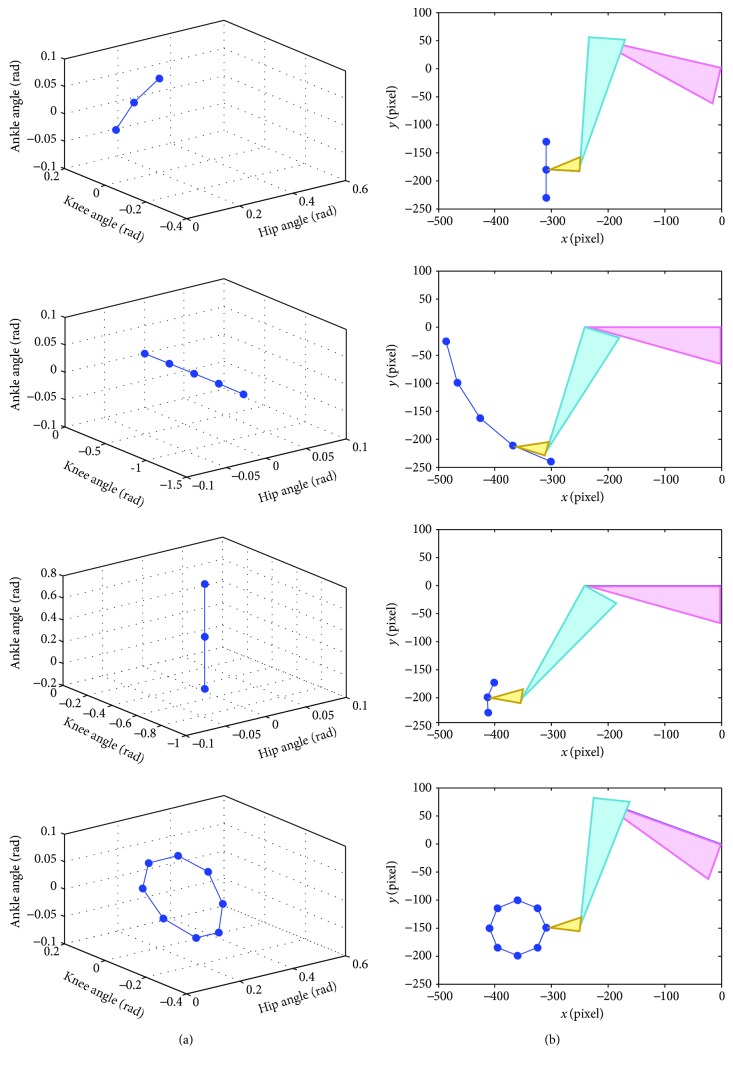
Reference paths for seated marching exercise, the single-joint training at knee and ankle joint, and cycling exercise in (a) joint space and (b) Cartesian space.

Before the training session, the reference path must be defined by teaching the robot. By manually moving the exoskeleton (and the subject's leg) to the starting point of the desired movement, the robot operator can use the user interface shown on the monitor screen to record the current joint position of the robot. The next points of the desired movement are also obtained while moving the exoskeleton and recording a sequence of the desired position. When the teaching is done, the reference path for the training is generated by connecting a series of the selected points with straight lines. The desired joint angles are linearly interpolated between a selected point and the consecutive point. The repetition of the path can be selected as moving back and forth or as a cycle (the last point connected to the first point). The desired path can be generated to perform various training activities both single-joint and multiple-joint training.  Figure 8 presents the reference path of seated marching exercise (for hip flexion exercise), the single-joint training at knee and ankle joint, and cycling exercise both in joint space and Cartesian space. The virtual leg of the subject is illustrated as triangles for thigh (pink), shank (blue), and foot (yellow) segments. It is displayed on the monitor screen as a visual feedback to the subject while tracking the reference trajectory.

Once the reference trajectory is generated, the operator must select the trajectory speed and assistance level. When the training starts, the reference position moves from the first point to the last point along the reference path and repeats the movement. Actual joint angle and torque are recorded at the frequency of 100 Hz and shown on the monitor screen. The training session continues until the “stop” button on the user interface is pressed.

In this study, a preliminary experiment was conducted on a volunteered healthy subject (male, age 28 years, weight 65 kg, height 168 cm, and without history of neurological disorder). The training activities include seated marching exercise, training at knee and ankle joints, and cycling training whose reference paths are shown in Figure 8. The subject is informed to keep tracking the reference trajectory, which is shown on a monitor screen along with the current position of the robot, as much as possible. Speed of the trajectories in Cartesian space is set as a constant throughout the training. However, for the single-joint training, when reaching the first and the last point of the reference path, the movement is paused for one second. Each training consists of 8 cycles of movement. Control gains *K*_1_, *K*_2_, and *K*_*d*_ used in this experiment are referred to Table 7.

### 5.2. Statistical Data Analysis

The data of the movement is separated into data from each cycle. Time spent on a cycle is normalized so that 0% represents the start of the cycle and 100% is the end of the cycle. Average angle $\left({\bar{\theta }}_{i\%}\right)$ and assistance torque $\left({\bar{T}}_{i\%}\right)$ at *i*% of a movement cycle are calculated from

(9) $$\begin{array}{c} {\bar{\theta }}_{i\%}=\frac{\sum_{j=1}^{n}\theta_{i\%}^{j}}{n},\\ {\bar{T}}_{i\%}=\frac{\sum_{j=1}^{n}T_{i\%}^{j}}{n}, \end{array}$$

where *n* is the number of movement cycles which is equal to 8 for this experiment, and *θ*_*i*%_^*j*^ and *T*_*i*%_^*j*^ are angle and torque at *i*% of the *j*^th^ cycle. The average data profile is obtained by connecting average angle from 0% to 100%.

The root mean square value is chosen as the representative of the average data in a movement cycle. The root mean square error $\left(e_{\theta_{ref}-\bar{\theta },rms}\right)$ between the reference trajectory (*θ*_ref_) and the average trajectory $\left(\bar{\theta }\right)$ is computed as follows:

(10) $$\begin{array}{c} e_{\theta_{ref}-\bar{\theta },rms}=\sqrt{\frac{\sum_{k=1}^{N}{\left(\theta_{ref,i\%}-{\bar{\theta }}_{i\%}\right)}^{2}}{N}}, \end{array}$$

where *N* is the number of data in one cycle of movement (index *k* = 1 and *k* = *N* refers to data at 0% and 100%). Note that the reference trajectory is the same in every movement cycle, so *θ*_ref,*i*%_ is not averaged.

To determine the deviation between the trajectory of each movement cycle and the average trajectory, another error that compares the angle of the *j*^th^ cycle to the average angle at *i*%$\left(e_{\theta -\bar{\theta },i\%}^{j}\right)$ is described by

(11) $$\begin{array}{c} e_{\theta -\bar{\theta },i\%}^{j}=\theta_{i\%}^{j}-{\bar{\theta }}_{i\%}^{j}. \end{array}$$

The root mean square error of the *j*^th^ cycle $\left(e_{\theta -\bar{\theta },rms}^{j}\right)$is

(12) $$\begin{array}{c} e_{\theta -\bar{\theta },rms}^{j}=\sqrt{\frac{\sum_{k=1}^{N}{\left(e_{\theta -\bar{\theta },i\%}^{j}\right)}^{2}}{N}}. \end{array}$$

The standard deviation of the root mean square error $\left(SD_{e_{\theta -\bar{\theta },rms}}\right)$ is calculated to identify the variation of data from 8 movement cycles:

(13) $$\begin{array}{c} {SD}_{e_{\theta -\bar{\theta },rms}}=\sqrt{\frac{\sum_{j=1}^{n}{\left(e_{\theta -\bar{\theta },rms}^{j}-{\bar{e}}_{\theta -\bar{\theta },rms}\right)}^{2}}{n}}, \end{array}$$

where

(14) $$\begin{array}{c} {\bar{e}}_{\theta -\bar{\theta },rms}=\frac{\sum_{j=1}^{n}e_{\theta -\bar{\theta },rms}^{j}}{n}. \end{array}$$

The root mean square average torque $\left({\bar{T}}_{rms}\right)$ is derived as follows:

(15) $$\begin{array}{c} {\bar{T}}_{rms}=\sqrt{\frac{\sum_{k=1}^{N}{\left({\bar{T}}_{i\%}\right)}^{2}}{N}}, \end{array}$$

where ${\bar{T}}_{i\%}$ is the torque averaged from 8 movement cycles at *i*%. The root mean square average torque is a good representation showing the amount of assistance torque provided to a subject because the value of positive and negative sign is not canceled out. The direction of the assistance torque can be observed from the average torque profile.

### 5.3. Results

#### 5.3.1. Seated Marching Exercise

**Figure 9:**
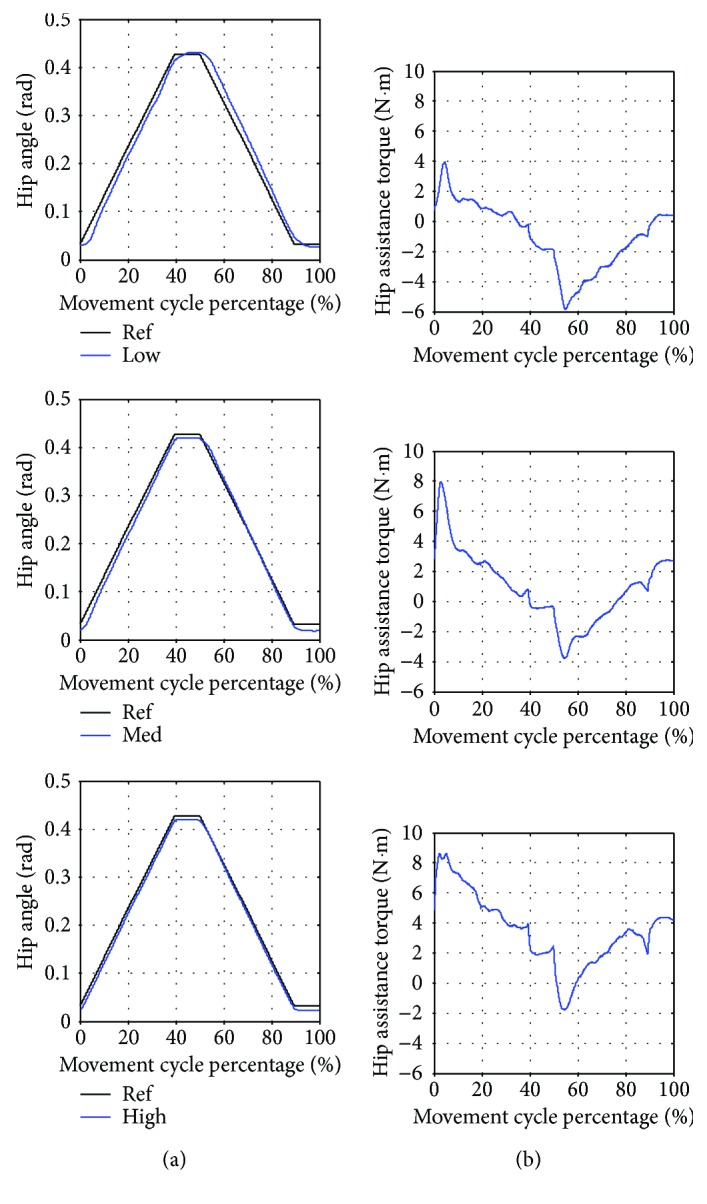
Seated marching exercise with low (Low), medium (Med), and high (High) assistance. (a) Average hip angle compared to the reference trajectory (Ref). (b) Average hip assistance torque.

In Figure 9, the average hip trajectory and torque obtained from 8 movement cycles and 3 different assistance levels are shown with respect to movement cycle percentage (0% and 100% represent the start and the end of each movement cycle). The movement starts by performing hip flexion (increasing hip angle) and pauses for one second (constant maximum hip angle) and then performing hip extension (decreasing hip angle) and pauses for another one second (constant minimum hip angle to complete the cycle). One cycle of the movement takes 9.45 seconds.

According to Table 8, the training with low assistance has the highest angle error. In Figure 9, the average trajectory of low assistance training has the largest deviation from the reference trajectory while the average trajectory of medium and high assistance training is closer to the reference trajectory, respectively. The variation of the actual trajectory in 8 movement cycles as compared to the average trajectory can be identified by the standard deviation. It is noticed from Table 8 that the highest variation is found in the training with low assistance. Moreover, the average assistance torque during the training with low assistance also has the lowest magnitude. It can also be seen in Figure 9(b) that the torque profiles in low, medium, and high assistance training are similar when compared at each percentage of the movement cycle.

#### 5.3.2. Training at Knee Joint

**Figure 10:**
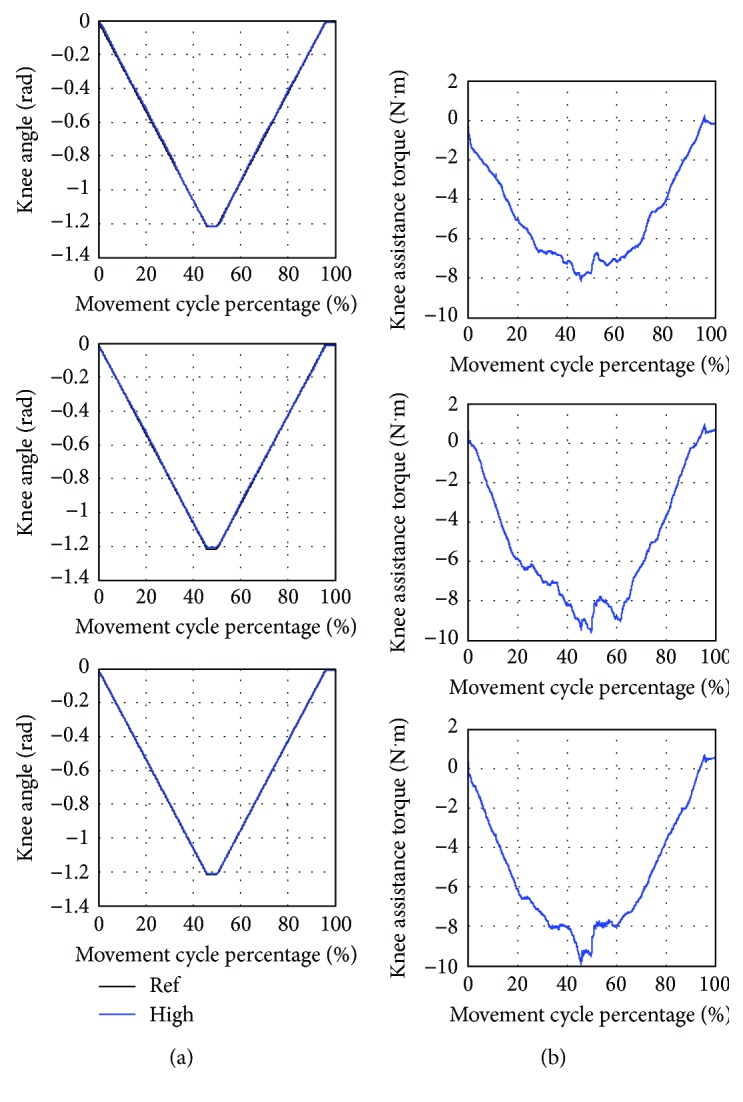
Training at knee joint with low (Low), medium (Med), and high (High) assistance. (a) Average knee angle compared to the reference trajectory (Ref). (b) Average knee assistance torque.

Figure 10 shows the average knee angle and torque during the training at knee joint. The movement starts by performing knee extension (decreasing knee angle) and pauses for one second (constant minimum knee angle) and then performing knee flexion (increasing knee angle) and ends after another one-second pause (constant maximum knee angle). Hip and ankle joints do not move, so their reference angles are fixed at zero. One cycle of the movement takes 24.43 seconds.

As shown in Table 9, the low assistance training has the highest error between the average and the reference trajectory. The standard deviation which shows variation of the actual trajectory in 8 movement cycles compared to the average trajectory is highest in the low assistance training. Besides, the lowest magnitude of average assistance torque is found in the low assistance training. As seen from Figure 10(b), the shapes of torque profiles are similar in low, medium, and high assistance training.

#### 5.3.3. Training at Ankle Joint

**Figure 11:**
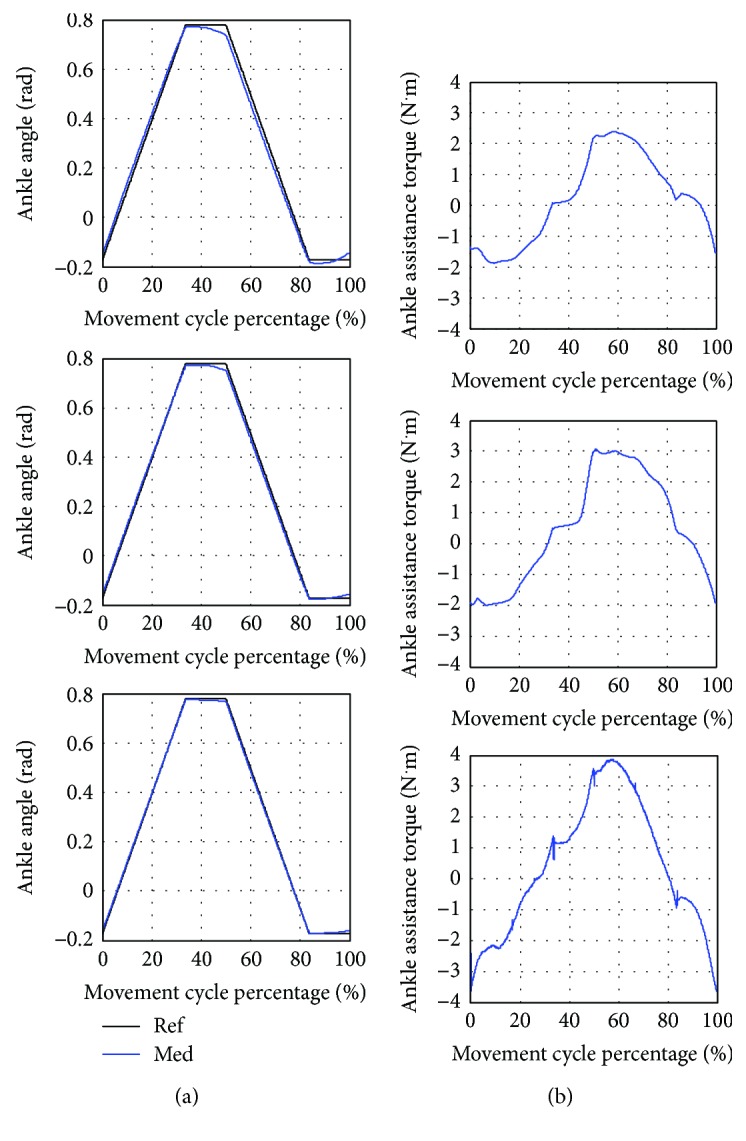
Training at ankle joint with low (Low), medium (Med), and high (High) assistance. (a) Average ankle angle compared to the reference trajectory (Ref). (b) Average ankle assistance torque.

The average ankle angle and torque during the training are shown in Figure 11. The movement starts from performing ankle plantar flexion (increasing ankle angle) and pauses for one second and then performing ankle dorsiflexion (decreasing ankle angle) and pauses for another one second. During the training, the knee angle is fixed at a negative constant angle to avoid the foot slapping on the floor. One cycle of the movement takes 6.13 seconds.

According to Table 10, the highest error between the average and the reference trajectory is found in the low assistance training. Large variation of the actual trajectory in 8 movement cycles compared to the average trajectory also occurs in the low assistance training. Moreover, the robot provides the lowest average assistance torque to the subject in low assistance training. It could be noticed that the variations of the movement in the medium and high assistance are similar. The torque profiles in low, medium, and high assistance as shown in Figure 11(b) are also resemblant.

#### 5.3.4. Cycling Exercise

**Figure 12:**
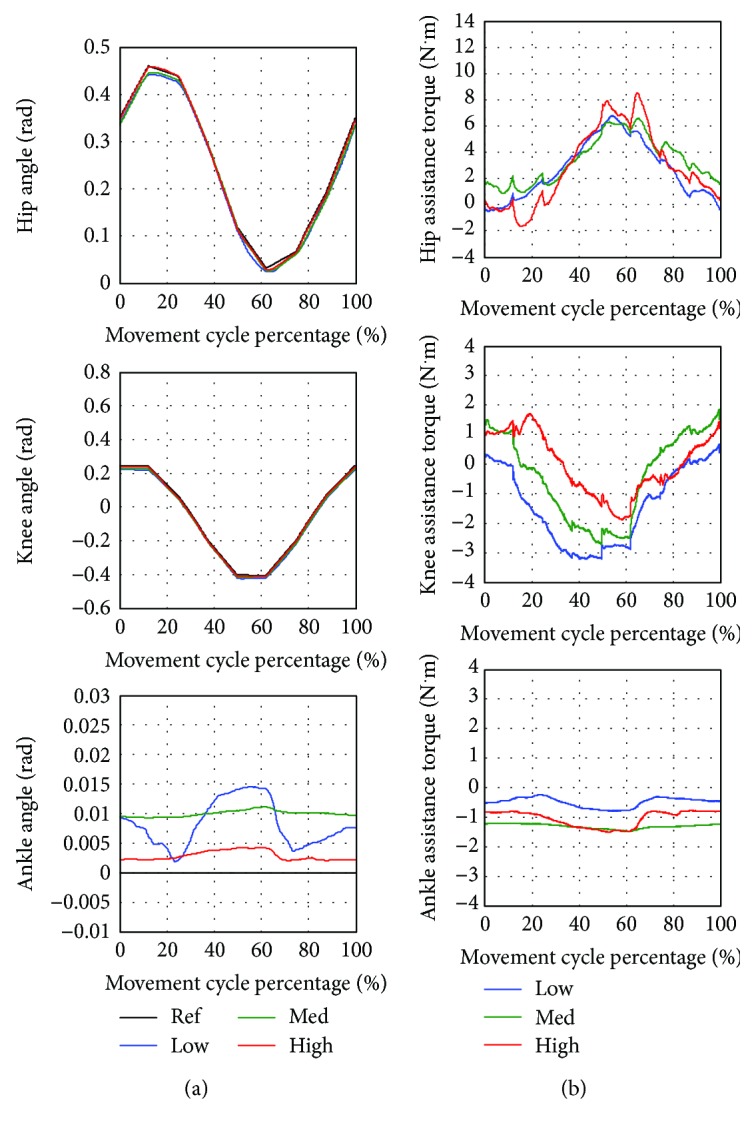
Cycling exercise with low (Low), medium (Med), and high (High) assistance. (a) Average hip, knee, and ankle angle compared to the reference trajectory (Ref). (b) Average hip, knee, and ankle assistance torque.

**Figure 13:**
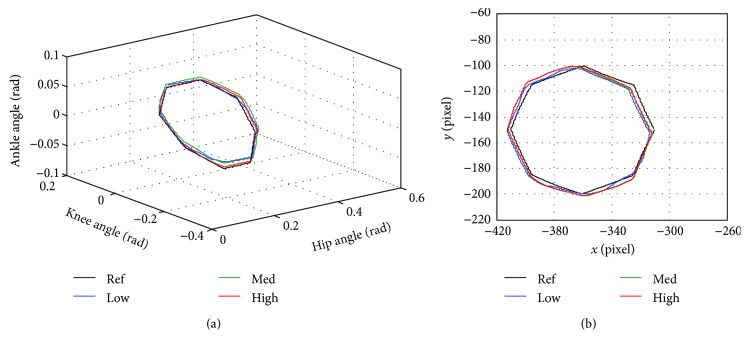
Cycling trajectory compared to the reference trajectory (Ref) with low (Low), medium (Med), and high (High) assistance in (a) joint space and (b) Cartesian space.

In Figure 12, the average angle and assistance torque of hip, knee, and ankle joints are compared when training with low, medium, and high assistance level. As noticed from Figure 13(b), the starting point of the movement is located at (*x*, *y*) = (−310 pixel, −150 pixel) and the direction of the movement is counterclockwise around the center of the circle located at (*x*, *y*) = (−360 pixel, −150 pixel). The cycling reference trajectory includes the movement of hip and knee joints while the ankle angle is fixed at zero. The reference trajectory is created from straight lines connecting reference points to the consecutive points. The movement is continuous, so there is no pause in a movement cycle. One cycle of movement takes 11.45 seconds.

As noticed from Table 11, the highest error between the average and reference trajectory almost occurs in the training with low assistance. High variation of the movement is also likely to be found in the low assistance training compared to the medium and high assistance training. Furthermore, the average assistance torque applied by the robot is usually low in the low assistance training while the medium and high assistance training tend to generate higher magnitude of assistance torque. It can be seen from Figure 12(b) that the shapes of torque profiles for low, medium, and high assistance are similar.  Figure 13 compares the average trajectory to the reference trajectory when the subject trained with low, medium, and high assistance. The average trajectories as seen in joint space and Cartesian space are closed to the reference trajectory with some degree of angle error.

#### 5.3.5. Discussion

The experiment has shown that the robot is able to train the subject with many activities and levels of assistance. The subject can track the reference trajectories with some angle errors. The magnitude of the error is usually high in low assistance training followed by medium and high assistance training.

The variation of the movement can be determined from the standard deviation which derived from the comparison between the actual trajectories in 8 movement cycles and the average trajectory. It was found that the low assistance training is likely to have the highest movement variation for hip, knee, and ankle joints in any training activities. In other words, the subject has more freedom to move on his own in the low assistance, even though the patterns of the movement in each cycles are not consistent.

The average magnitude of assistance torque is usually lowest in the low assistance training. Shapes of the torque profiles for low, medium, and high assistance are similar when comparing at each movement cycle percentage. It could be seen that there are abrupt changes of the torque profile in the seated marching exercise and the single-joint training at knee and ankle joints at the transitions before and after the movement pauses. These might result from the speed of the trajectory which is set as a constant and absence of smooth changes at these transitions. Assistance torque changes rapidly in order to create acceleration/deceleration for stopping or initiating the movement. These abrupt changes are also found in the cycling exercise when changing the reference point. Although the speed remains constant, the direction of the movement changes at the reference points. Thus, assistance torque changes suddenly in order to create acceleration for changing the direction of the movement at these transitions.

## 6. Conclusion

Lower limb rehabilitation robots in sitting position have been researched extensively. Rehabilitation robots were developed into many types and targeted at different degrees of freedom for the physical therapy. Training activities performed by these robots differ according to robot's configuration and the selection of training modalities. These activities can be categorized as single-joint and multiple-joint training. The single-joint training focuses on the movement of an individual joint such as hip, knee, or ankle joint. On the other hand, the multiple-joint training associates the movement of many joints in the same time so that a variety of exercises such as leg press, cycling, gait trajectory following, or customized movement could be performed. Some robots were developed to perform a specific training activity while the others are able to perform several training activities.

A lower limb rehabilitation robot in sitting position for stroke patients was developed in the previous research. It has three degrees of freedom at hip, knee, and ankle joints which allow movements of lower limbs in sagittal plane. This robot is able to perform many training activities and modalities. The control system for active assistive exercise is described in detail. The impedance control law implemented by the developed rehabilitation robot uses two constants to define the relationship between angle error and desired torque to be generated by a robot joint. With the damping term in the impedance control law, the passivity property of the system is verified. These control gains are chosen based on the initial rate of change of desired torque, maximum allowable angle error and torque. Different sets of control gains result in different robot response due to disturbance torque. The robot is also tested under impact force to prove its robustness. The experiment conducted on a healthy subject has shown that the robot is able to perform many training activities such as seated marching exercise, single-joint training at knee and ankle joints, and cycling exercise with active assistive training modality and with many levels of assistance. It is found that low assistance training usually produces the highest error between the average trajectory and the reference trajectory. This implies that the subject is not restricted to move exactly along the reference trajectory. The standard deviation is derived by comparing the movement in each cycle to the average trajectory so that the variation of the movement could be investigated. The greatest movement variation is likely to be found in low assistance training than in medium and high assistance training. High angle deviation and movement variation in low assistance training imply that the subject could move the limbs with more freedom. The assistance torque is provided by the robot to ensure the completion of the movement. It is also found that the low assistance training usually generates the lowest magnitude of the assistance torque. Abrupt changes in assistance torque, which can be noticed in each training activity, result from the rapid change in speed and direction of the reference trajectory.

In future research, the movement of the robot at the transitions should be improved by smoothing the change in speed and direction at the transitions. Clinical trials should be conducted on stroke patients to verify the effectiveness of the robot and control system for stroke rehabilitation task.

## Figures and Tables

**Table 1:** Training activities of stroke rehabilitation robot in sitting/lying position.

Robot	Robot type	Actuated DOFs	Training activities	Training modalities
Anklebot	Foot robotic orthosis	Ankle dorsiflexion/plantar flexion, inversion/eversion	Training at ankle joint	Active assistive
Vi-RABT	Footplate-based end-effector robot	Ankle dorsiflexion/plantar flexion, inversion/eversion	Training at ankle joint	Active assistive, active
Ankle rehabilitation robot by Yoon et al.	Footplate-based end-effector robot	Ankle dorsiflexion/plantar flexion, inversion/eversion, metatarsophalangeal flexion/extension	Training at ankle joint, gait trajectory following	Passive, active assistive, active resistive
Lower limb paediatric therapy device by Chrif et al.	End-effector robot	Movement in sagittal plane	Leg press	Active resistive
Horizontal lower limb rehabilitation training robot by Guo et al.	End-effector robot	Movement in sagittal plane, ankle internal/external rotation	Training at single/multiple joints	Passive
ViGRR	End-effector robot	Movement in sagittal plane	Gait trajectory following, leg press	Active resistive
MOTOmed	End-effector robot	Movement in sagittal plane	Cycling	Passive, active assistive, active resistive
Physiotherabot	Exoskeleton	Hip flexion/extension, abduction/adduction, knee flexion/extension	Training at single/multiple joints, customized movement	Passive, active assistive, active resistive
Lower limb rehabilitation robot in our previous research	Exoskeleton	Hip flexion/extension, knee flexion/extension, ankle flexion/extension	Training at single/multiple joints, customized movement	Passive, active assistive, active resistive

**Table 2:** Training modalities implemented in each training activity.

Training activity	Training modalities			
	Passive	Active assistive	Active	Active resistive
Single-joint training (at hip, knee, or ankle joint)	*x*	*x*	*x*	*x*
Multiple-joint training				
Leg press				*x*
Cycling	*x*	*x*		*x*
Gait trajectory following				*x*
Customized movement		*x*		

**Table 3:** Specifications of brushless servomotors.

Power (W)	Peak stall torque (N·m)	Rated torque (N·m)	Rated speed (rpm)	Inertia (×10^−4^ kg · m^2^)
400	4.8	1.27	3000	0.412
200	2.2	0.637	3000	0.219

**Table 4:** Specifications of the robot joints.

Joint	Transmission ratio	Continuous torque (N·m)	Reflected motor inertia (kg · m^2^)
Hip	57.76 : 1	73.36	0.137
Knee	15 : 1	19.05	9.27 × 10^−3^
Ankle	15 : 1	9.56	4.93 × 10^−3^

**Table 5:** Dimensions and inertia properties of the robot segments.

Segment	Length (mm)	Moment of inertia about the proximal joint axis (kg · m^2^)
Thigh	365–465	0.141–0.258
Shank	365–465	0.140–0.259
Foot	75–95	0.006–0.007

**Table 6:** Impedance control gains of the ankle joint.

Controller	Initial rate of change of desired torque	Angle error at the maximum torque (rad)	*K*_1_	*K*_2_	*K*_*d*_
A	30	0.07	0.81	37.01	2.0
B	100	0.05	3.98	25.13	2.0
C	300	0.03	43.42	6.91	2.0

**Table 7:** Impedance control gains for hip, knee, and ankle joints in low, medium, and high assistance mode.

Joint	Low assistance mode			Medium assistance mode			High assistance mode		
	*K*_1_	*K*_2_	*K*_*d*_	*K*_1_	*K*_2_	*K*_*d*_	*K*_1_	*K*_2_	*K*_*d*_
Hip	0.44	67.62	5.0	1.38	72.30	5.0	3.20	93.67	5.0
Knee	0.59	50.71	4.0	2.14	46.73	4.0	6.29	47.65	4.0
Ankle	0.81	37.01	2.0	3.98	25.13	2.0	43.42	6.91	2.0

**Table 8:** Statistical data of the hip joint from the seated marching exercise.

Level of assistance	$e_{\theta_{ref}-\bar{\theta },rms}$ (rad)	${SD}_{e_{\theta -\bar{\theta },rms}}$ (×10^−4^ rad)	${\bar{T}}_{rms}$ (N·m)
Low	0.0198	28.31	2.40
Medium	0.0123	11.04	2.57
High	0.0090	6.55	4.25

**Table 9:** Statistical data from the training at knee joint.

Level of assistance	$e_{\theta_{ref}-\bar{\theta },rms}$ (rad)	${SD}_{e_{\theta -\bar{\theta },rms}}$ (×10^−4^ rad)	${\bar{T}}_{rms}$ (N·m)
Low	0.0107	23.26	5.35
Medium	0.0077	16.97	5.97
High	0.0033	9.00	6.05

**Table 10:** Statistical data from the training at ankle joint.

Level of assistance	$e_{\theta_{ref}-\bar{\theta },rms}$ (rad)	${SD}_{e_{\theta -\bar{\theta },rms}}$ (×10^−4^ rad)	${\bar{T}}_{rms}$ (N·m)
Low	0.0253	19.56	1.4
Medium	0.0141	4.42	1.81
High	0.0071	5.00	2.15

**Table 11:** Statistical data from the cycling training.

Statistical data	Low assistance			Medium assistance			High assistance		
	$e_{\theta_{ref}-\bar{\theta },rms}$ (rad)	${SD}_{e_{\theta -\bar{\theta },rms}}$(×10^−4^ rad)	${\bar{T}}_{rms}$ (N·m)	$e_{\theta_{ref}-\bar{\theta },rms}$ (rad)	${SD}_{e_{\theta -\bar{\theta },rms}}$(×10^−4^ rad)	${\bar{T}}_{rms}$ (N·m)	$e_{\theta_{ref}-\bar{\theta },rms}$ (rad)	${SD}_{e_{\theta -\bar{\theta },rms}}$(×10^−4^ rad)	${\bar{T}}_{rms}$ (N·m)
Hip	0.0125	25.31	3.50	0.0104	8.76	3.82	0.0041	9.14	4.11
Knee	0.0199	29.76	1.91	0.0150	6.91	1.50	0.0073	2.82	1.02
Ankle	0.0090	13.09	0.52	0.0100	1.29	1.31	0.0030	2.82	1.08
